# Comparative analysis of four sequencing platforms for methylation sequencing

**DOI:** 10.1186/s13104-026-07690-0

**Published:** 2026-02-16

**Authors:** Jinxia Guan, Chuanchun Yang, Hongjun Gao, Shuanping Liu, Min Zheng, Yongjun Li, Baochen Du, Changling Zhang, Yuanyuan Guo, Qi Wang, Luyang Zhao, XiaoLiang Han

**Affiliations:** 1 BioChain(Beijing) Science & Technology, Inc., Beijing, 102600 China; 2 Shanghai Salus Life Sciences Co., Ltd., 4 Floor, Building 6, No. 830 Chengyin Road, Dachang Town, Baoshan District, Shanghai, China; 3 Department of Clinical Laboratory, Shanxi Province Cancer Hospital, Shanxi Hospital Affiliated to Cancer Hospital, Chinese Academy of Medical Sciences, Cancer Hospital Affiliated to Shanxi Medical University, Taiyuan, 030013 China; 4 Respiratory and Critical Care Medicine, Emergency General Hospital & National Research Center for Emergency Medicine, Beijing, China

**Keywords:** Multiple sequencing platforms, DNA methylation, CpG

## Abstract

This study conducts a comprehensive assessment of four sequencing platforms: GenoLab M (Genemind), NovaSeq X (Illumina), T7 (MGI) and Salus Pro (Salus), focusing on different performance metrics related to methylation data. By analyzing data from plasma cfDNA samples, we assessed key metrics including sequencing quality, CpG coverage, data efficiency, and methylation level consistency. These findings aim to provide valuable insights into the consistency of sequencing platforms for researchers and laboratories when integrating multiple methylation datasets for analysis.

## Highlights

- Three sequencing platforms demonstrate comparable performance on methylation data.
- The MGI platform exhibits a higher overall methylation level in the target region relative to the other three platforms.
- Ensuring consistent assessment across different platforms is essential when combining datasets.

## Introduction

DNA methylation data have emerged as a valuable biomarker source, as it dynamically varies with exogenous/endogenous factors (e.g., environmental risks and disease pathology), unlike static genetic risk profiles [1]. More importantly, with advancements in genomic and molecular technologies, circulating cell-free DNA (cfDNA) has huge potential as a promising prognostic and predictive biomarker in liquid biopsy applications [2]. In the field of methylation analysis, accurate and efficient sequencing is crucial for a wide range of applications, from basic research to clinical diagnosis [3]. With the development of sequencing technologies, multiple platforms have emerged, each with its unique features [4, 5]. In this research, we focus on four widely used sequencing platforms, GenoLab M (Genemind), NovaSeq X (Illumina), T7 (MGI) and Salus Pro (Salus), and conduct a detailed performance evaluation. To the best of our knowledge, this is the first comparative analysis of multiple platforms for methylation sequencing.

The platforms Genemind, Illumina, and Salus all utilize bridge PCR technology for sequencing-by-synthesis (SBS), a core component of their sequencing workflows. In this process, DNA fragments are anchored to a solid substrate, where primer hybridization and iterative cycles of denaturation, annealing, and extension generate clonal DNA clusters. These clusters amplify signal intensity, enabling precise base calling during the SBS reaction [6]. Since the sequencing principles that they all rely on shared workflow steps—including DNA fragmentation, adapter ligation, and amplification—libraries can be used interchangeably across these platforms provided that they adhere to the fundamental requirements of bridge PCR-based sequencing systems [7].

In contrast, the platform MGI employs the Combinatorial Probe-Anchor Synthesis (cPAS) method, which relies on DNA nanoballs (DNBs) as its core sequencing architecture [8]. Additionally, to adapt libraries for platform MGI, the workflow involves transforming double-stranded Illumina libraries into DNBs through a precise enzymatic process.

The comparison of these platforms helps in understanding their strengths and limitations, enabling better decision-making in experimental design and data interpretation.

## Materials and methods

### Sample collection and preparation

Three volunteers were recruited for this project, each of whom signed an informed consent form. Peripheral blood was drawn, from which cfDNA was isolated using the QIAGEN cfDNA Extraction Kit (Cat. No.55114, QIAGEN), yielding sufficient quantities of cfDNA. The samples were designated as SA-0014, SA-0015, and SA-0016, respectively.

### Library preparation

To minimize experimental variables and enhance the robustness of comparisons, a single library was constructed for each sample. This library was then equally partitioned into four sub-libraries, which were individually subjected to sequencing on the four sequencer platforms.

For platforms Genemind, Illumina, and Salus, cfDNA was subjected to bisulfite conversion (D5005, ZYMO RESEARCH), transforming unmethylated cytosines to uracils while preserving methylated cytosines. Bisulfite-treated cfDNA underwent ligation to adapters and was prepared as a dual indexed sequencing library. Library fragments were amplified using KAPA HiFi HotStart Uracil + ReadyMix (KAPA Biosystems). The product was enriched using the hybridization capture kit (Twist Biosciences, San Francisco, CA). Final libraries were purified via AMPure XP beads (1.0 ×) and quantified with QuBit, followed by paired-end 150 (PE150) sequencing. The targeted methylation panel covered 90,371 distinct regions (32.1 Mb) and 2,506,844 CpGs.

For platform MGI, to comply with the library requirements of the Salus sequencer, one sub-library was selected from four candidates for parallel conversion into a DNB library. Following its DNB library preparation protocol, denaturation and circularization steps were applied to convert the sub-libraries of three samples into DNB libraries, respectively.

### Sequencing process

The sequencing operations on each platform were carried out according to the recommended procedures. Each platform has its own specific sequencing chemistry and instrument settings. For all four sequencers, we employed the PE150 sequencing strategy.

### Data analysis

To eliminate performance variations caused by unequal data volumes, we randomly subsampled 30 GB of raw data from each sequencer's output for every sample using the SeqKit2 sample tool [9]. All downstream analyses were conducted using a unified analytical pipeline on these standardized datasets, ensuring consistency and comparability across experimental groups [10]. Firstly, the standardized raw data underwent quality evaluation using fastp (v0.21.0) to assess sequencing metrics (e.g., base quality, adapter contamination, Q20, Q30, and GC content distribution) [11]. Then, all reads were mapped to the converted genomes (hg19), and subsequent deduplication was performed using BisMark (v0.23) [12]. The deduplicated reads were used for calculating methylation levels by Bismark. Finally, mapping Quality-related metrics such as Mapping Rate (MapRate), Duplication Rate (DupRate), depth, Sequencing Coverage (Coverage), and Bisulfite Conversion Rate (BSRate) were calculated. MapRate is calculated as the number of reads uniquely aligned to the reference genome divided by the total number of clean reads, reflecting data efficiency. DupRate is calculated as the number of PCR amplification introduced redundant reads (identified using deduplicate-bismark) divided by the total number of mapped reads, with a lower value indicating less PCR bias. Depth represents the average effective depth in the target region, and a higher depth implies better data efficiency. Coverage is defined as the proportion of bases detected at least once within the target interval. BSRate is calculated as the total number of C reads divided by the total number of C reads and T reads for non-CpG cytosines, reflecting the bisulfite conversion efficiency. In addition, CpG site coverage, that is CpG detection rate, is defined as the proportion of targeted CpG sites covered by at least 1 read. Methylation level is calculated as the total number of C reads divided by the total number of C reads and T reads.

## Results

### Sequencing quality

The average sequencing data size across the four platforms is 42.9 Gb (31.0 Gb ~ 59.0 Gb, with a median of 41.0 Gb; Table 1). Salus achieved an average effective depth of 339.3X across three samples (~ 11.31X/Gb for the subsampled dataset; Table 3), outperforming Genemind (313X, ~ 10.43X/Gb), MGI (266.7X, ~ 8.89X/Gb), and Illumina (261.7X, ~ 8.72X/Gb) by ~ 11.8%, 27.2%, and 29.7% respectively. For parallel comparison, we randomly subsampled 30 Gb of reads from each dataset to eliminate biases caused by unequal data volumes. The Salus platform exhibited superior performance in key technical metrics related to sequencing quality, alignment rate, and data efficiency. As expected, all platforms showed substantial consistency in insert size and GC content (Table 2), which is attributed to their use of the same parent library for sequencing.

**Table 1:** Raw data statistics for 4 sequencing platforms

Platforms	Genemind			Illumina			MGI			Salus		
Samples	SA-0014	SA-0015	SA-0016	SA-0014	SA-0015	SA-0016	SA-0014	SA-0015	SA-0016	SA-0014	SA-0015	SA-0016
Total reads [million]	343	391	366	252	241	226	209	332	248	280	278	268
PE reads length [bp]	150	150	150	150	150	150	150	150	150	150	150	150
Total bases [Gbp]	51	59	55	38	36	34	31	50	37	42	42	40
Subset bases [Gbp]	30	30	30	30	30	30	30	30	30	30	30	30
Panel size [Mbp]	32	32	32	32	32	32	32	32	32	32	32	32

**Table 2:** Subdataset metrics for 4 sequencing platforms

Platforms	Genemind			Illumina			MGI			Salus		
Samples	SA-0014	SA-0015	SA-0016	SA-0014	SA-0015	SA-0016	SA-0014	SA-0015	SA-0016	SA-0014	SA-0015	SA-0016
Subset bases [Gbp]	30	30	30	30	30	30	30	30	30	30	30	30
Insert size [bp]	161	161	163	161	161	163	155	153	153	158	157	159
GC%	32.9	33.1	33.2	33.8	34.0	34.0	34.5	36.0	35.3	33.2	33.4	33.4
Q20	0.954	0.955	0.953	0.970	0.971	0.969	0.982	0.984	0.983	0.987	0.987	0.987
Q30	0.887	0.891	0.886	0.917	0.920	0.917	0.952	0.955	0.953	0.920	0.921	0.920

**Fig. 1:**
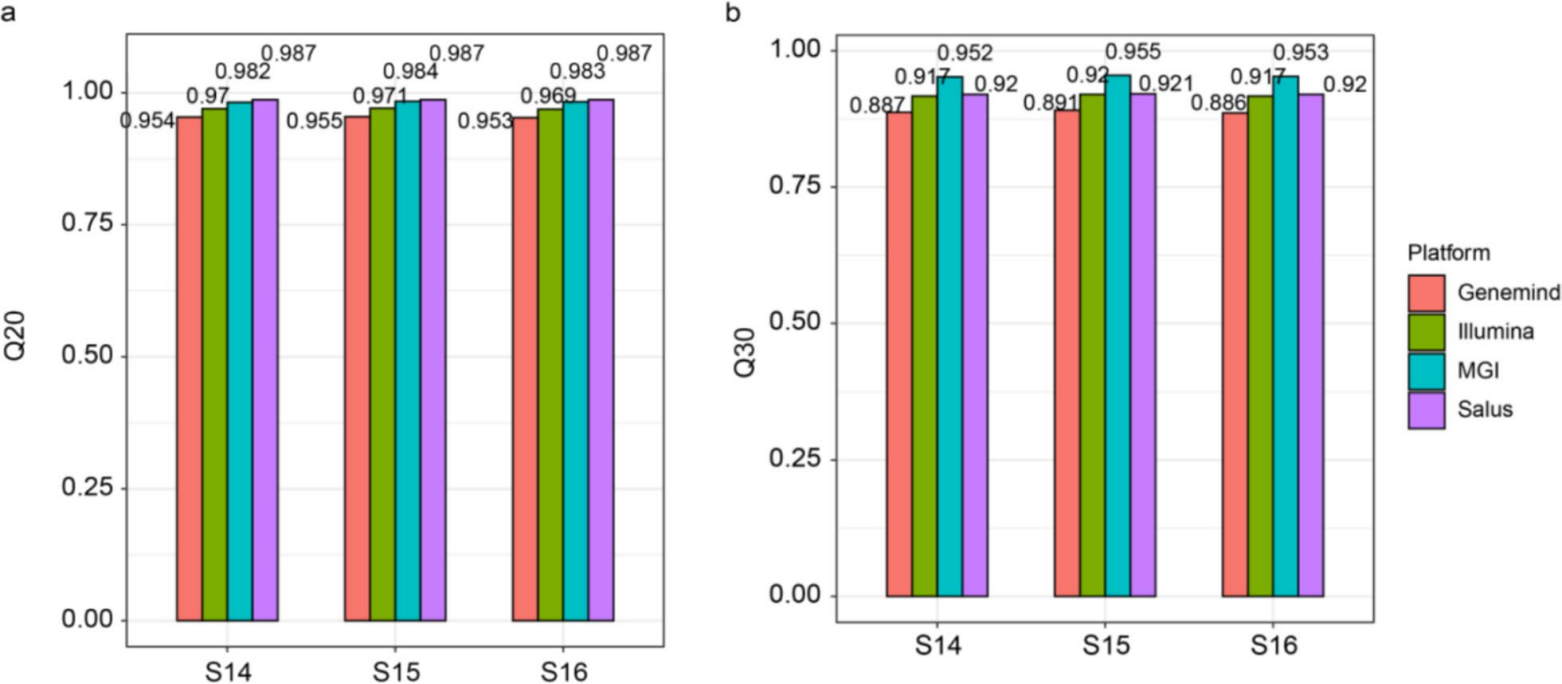
Base quality of the subsampled data across four platforms based on the Q20 (**a**) and Q30 (**b**) metrics

In terms of Q20 (a critical indicator of base-calling accuracy), the Salus platform achieved an average value of 98.7% across three samples, which was 1.7% higher than Illumina (average: 97.0%) and 3.3% higher than Genemind (average: 95.4%)—and comparable to MGI (average: 98.3%; Fig. 1a). For Q30 (a stricter measure of high-quality bases), the Salus platform reached an average of 92.0%, nearly identical to Illumina (average: 91.8%) and 3.2% higher than Genemind (average: 88.8%). MGI exhibited the highest average Q30 value at 95.3% among the four platforms (Fig. 1b).

### Mapped quality

**Fig. 2:**
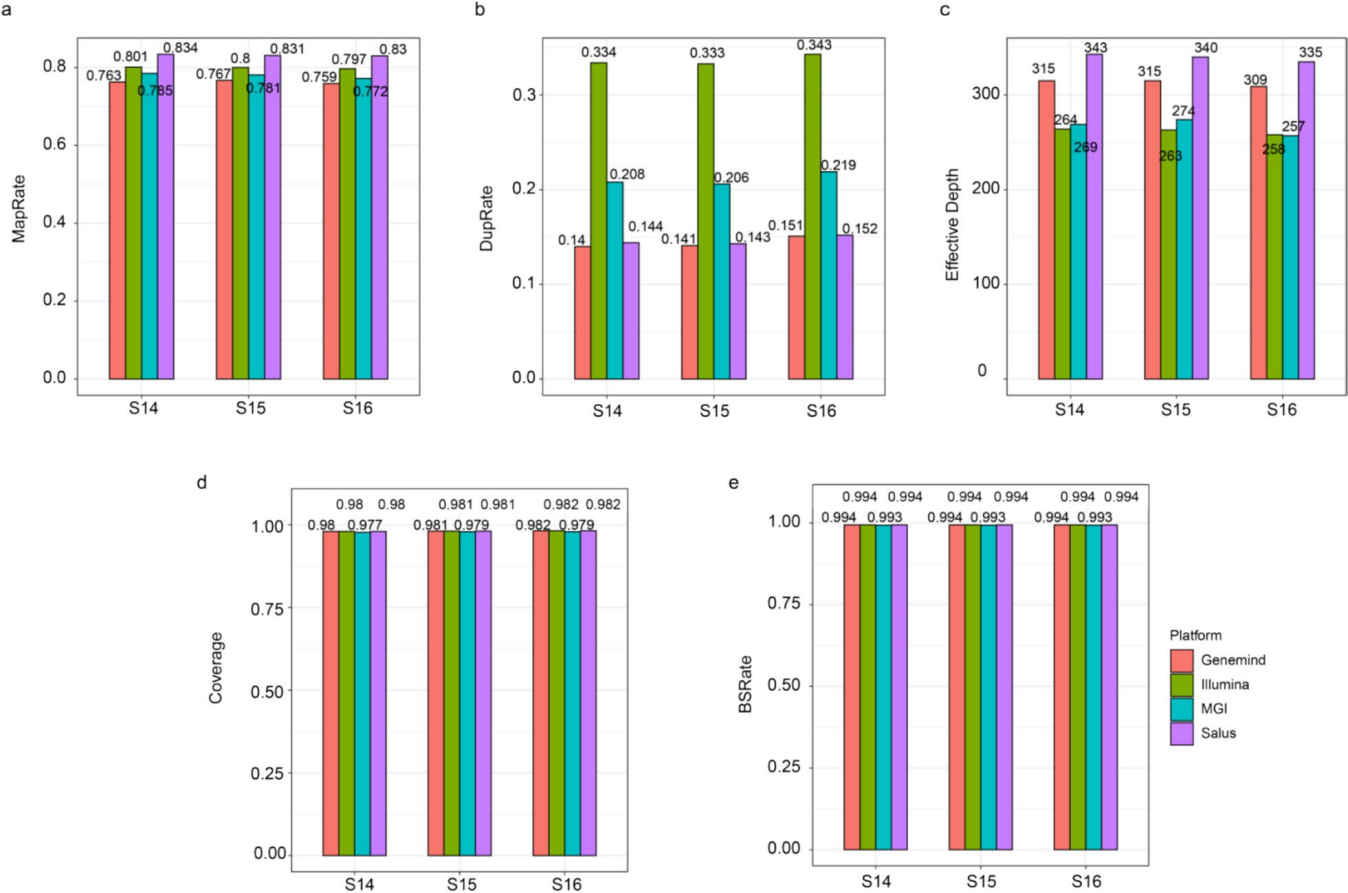
Comparisons of sequencing metrics using subsampled data across four platforms, including Mapping Rate (MapRate) (**a**), Duplication Rate (DupRate) (**b**), effective depth (**c**), Sequencing Coverage (Coverage) (**d**), and Bisulfite Conversion Rate (BSRate) (**e**)

In terms of MapRate, the Salus platform outperformed Illumina, MGI and Genemind by 3.2%, 6.7%, and 6.9%, respectively (Fig. 2a). For DupRate, the Salus platform was comparable to platform Genemind, but exhibited 30.8% and 56.7% reduction compared to MGI and Illumina (Fig. 2b). Regarding effective depth, platform Salus demonstrated a 7.9%, 27.2%, and 29.4% advantage over Genemind, MGI, and Illumina (Fig. 2c). In coverage and BSRate, the Salus platform showed no significant differences from Illumina and Genemind. All three platforms achieved a BSRate of 0.99, indicating equivalent data quality in terms of sequencing uniformity and bisulfite conversion efficiency (Fig. 2e). The platforms Genemind, Illumina, and Salus exhibited a coverage rate of 0.981 (Fig. 2d) and the MGI platform showed a coverage rate of 0.979 (Table 3).

**Table 3:** Mapped quality for 4 sequencing platforms

Platforms	Genemind			Illumina			MGI			Salus		
Samples	SA-0014	SA-0015	SA-0016	SA-0014	SA-0015	SA-0016	SA-0014	SA-0015	SA-0016	SA-0014	SA-0015	SA-0016
Subset bases [Gbp]	30	30	30	30	30	30	30	30	30	30	30	30
MapRate	0.763	0.767	0.759	0.801	0.800	0.797	0.785	0.781	0.772	0.834	0.831	0.83
DupRate	0.140	0.141	0.151	0.334	0.333	0.343	0.208	0.206	0.219	0.144	0.143	0.152
Effective Depth	315	315	309	264	263	258	269	274	257	343	340	335
Coverage	98.0%	98.1%	98.2%	98.0%	98.1%	98.2%	97.7%	97.9%	97.9%	98.0%	98.1%	98.2%
BSRate	0.994	0.994	0.994	0.994	0.994	0.994	0.993	0.993	0.993	0.994	0.994	0.994
CpG detection Rate^#^	96.3%	96.5%	96.8%	96.4%	96.6%	96.9%	91.2%	91.4%	91.9%	96.3%	96.4%	96.7%

^#^Defined as the proportion of targeted CpG sites covered by at least 1 read

### CpG detection rate

**Fig. 3:**
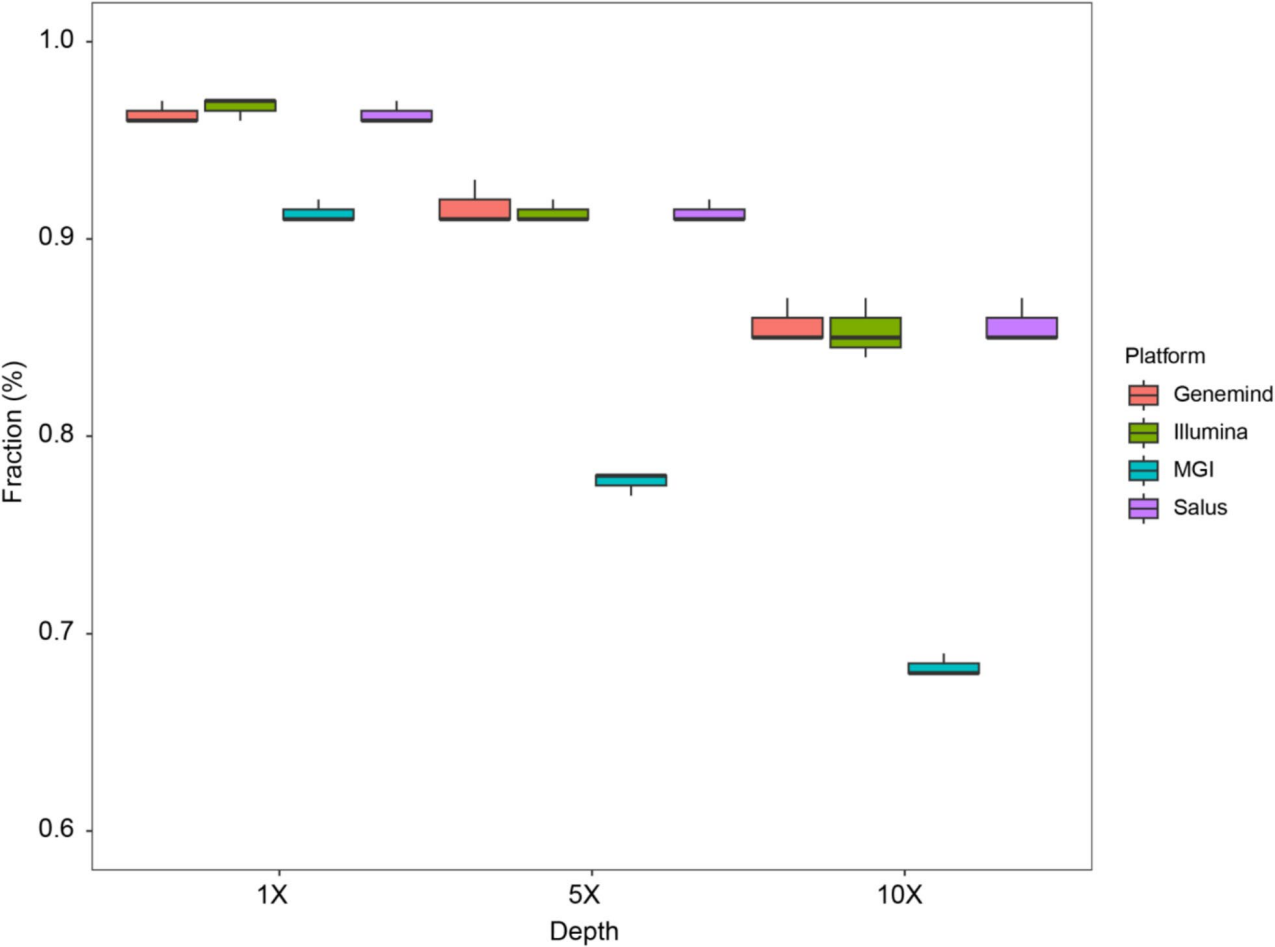
CpG detection rates across four sequencing platforms based on 1X read, 5X reads and 10X reads, respectively. The boxplots present downsampled 30G data from three patients

The platforms Genemind, Illumina, and Salus had similar CpG detection rates, all higher than that of MGI, regardless of whether the read coverage threshold was set to a low level (1X) or a high level (10X). The CpG detection rates of Genemind, Illumina, and Salus were greater than 90% (at 5X), while that of the MGI platform was less than 80% (at 5X). The platforms Genemind, Illumina, and Salus identified approximately 17.3% more CpGs with at least 5 reads compared to MGI (Fig. 3).

### Methylation level consistency

**Fig. 4:**
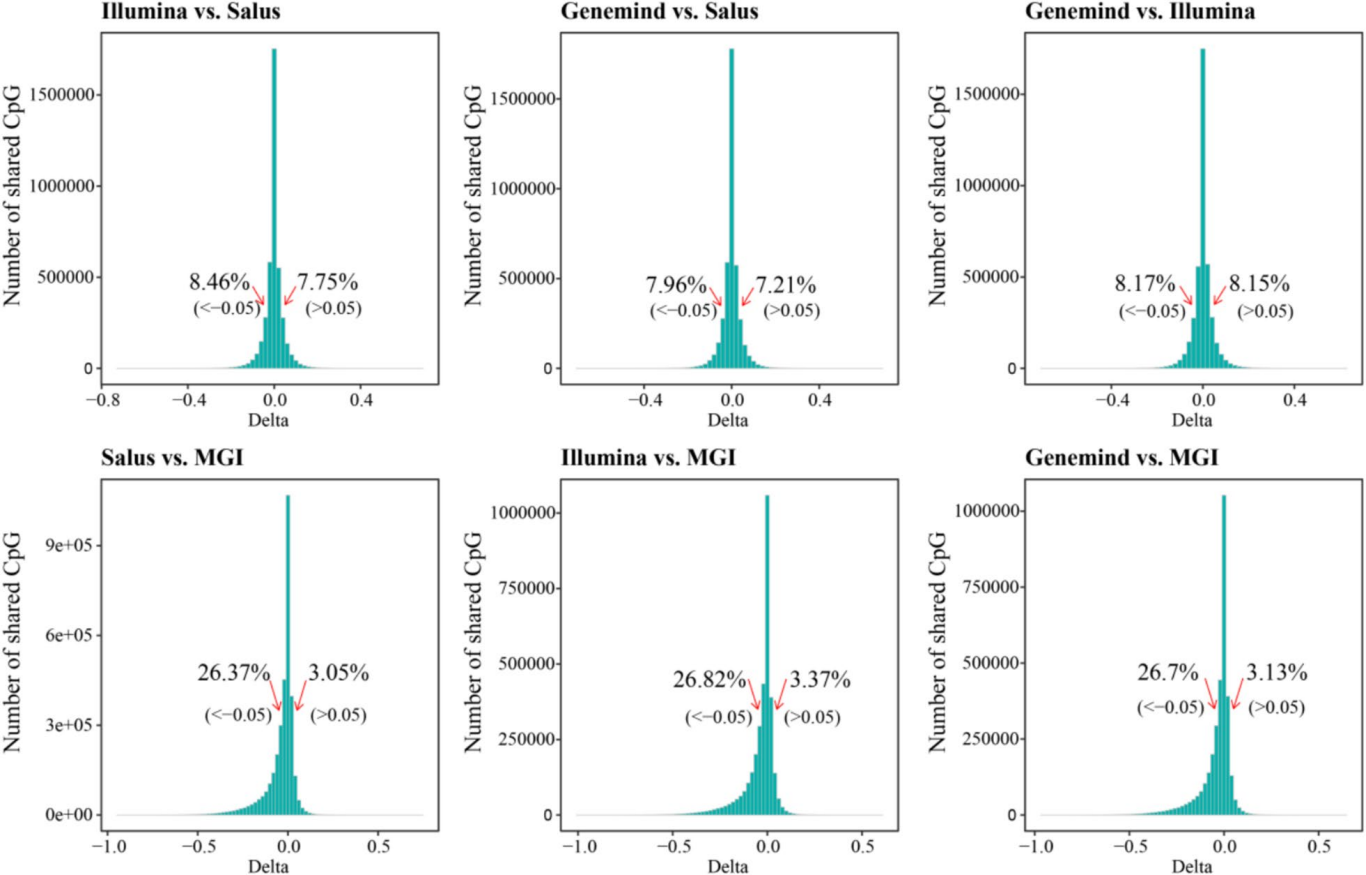
Comparisons of methylation difference (delta) between two platforms for sample 14. The red arrows indicate the percentage of CpGs that have absolute methylation differences exceeding 0.05. The left arrow represents hypomethylation, and the right arrow represents hypermethylation

**Fig. 5:**
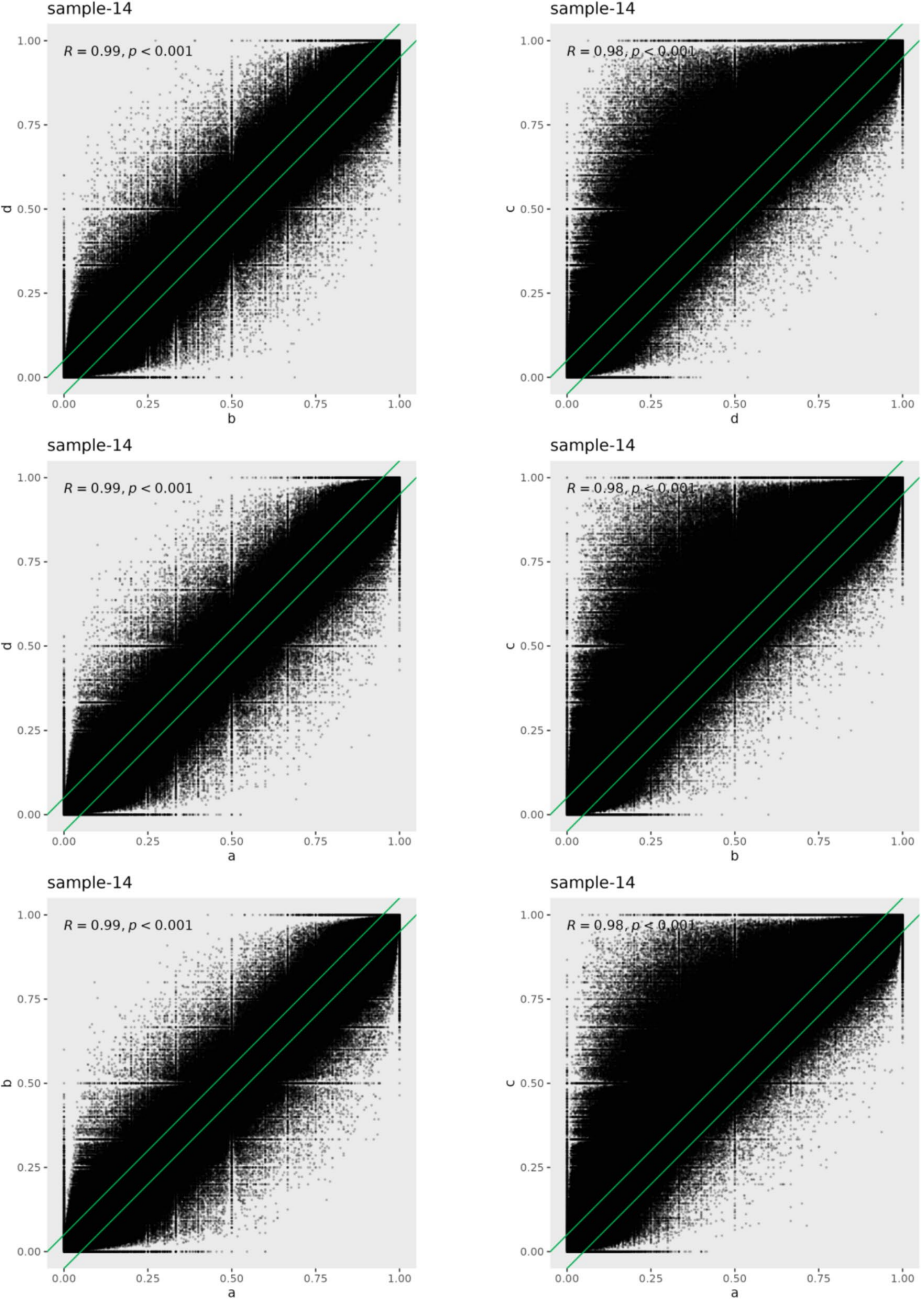
The consistency of methylation levels at individual CpGs across two different sequencing platforms. The points represent CpGs detected by both platforms. Top left presentes the Pearson's correlation coefficients and p values for the CpG methylation levels, as measured by two distinct platforms. The green line represents CpGs that have an absolute delta of 0.05. Labels a, b, c, and d correspond to the Genemind, Illumina, MGI, and Salus platforms, respectively

To evaluate the consistency of methylation at CpG sites across four platforms, we performed differential methylation analysis for Sample 14, comparing each pair of platforms. The differentially methylated CpGs (DMCs) revealed two distinct patterns (|delta|> 0.05). The first pattern indicated that approximately15.9% of DMCs were identified among platforms Genemind, Illumina, and Salus, with similar proportions of hypermethylated and hypomethylated CpGs. The other pattern showed that about 29.8% of DMCs were identified between platforms Genemind, Illumina, Salus and the MGI platform, with a notable 26.6% of DMCs exhibiting hypermethylation in the MGI platform (Fig. 4). When comparing the MGI platform with other platforms, it had 18.9% more hypermethylated and 5% fewer hypomethylated CpGs, suggesting that methylation levels of CpG sites varied for the MGI platform. (Fig. 5). Specifically, comparisons of the mean overall methylation levels across the four platforms revealed that the mean methylation levels of the Genemind, Illumina, and Salus platforms in the three samples were comparable, ranging from 44.6% to 46.6%. In contrast, the MGI platform exhibited a markedly higher overall methylation level (66.8%), which was greater than those of the other three sequencing platforms.

### DMCs evaluation

**Fig. 6:**
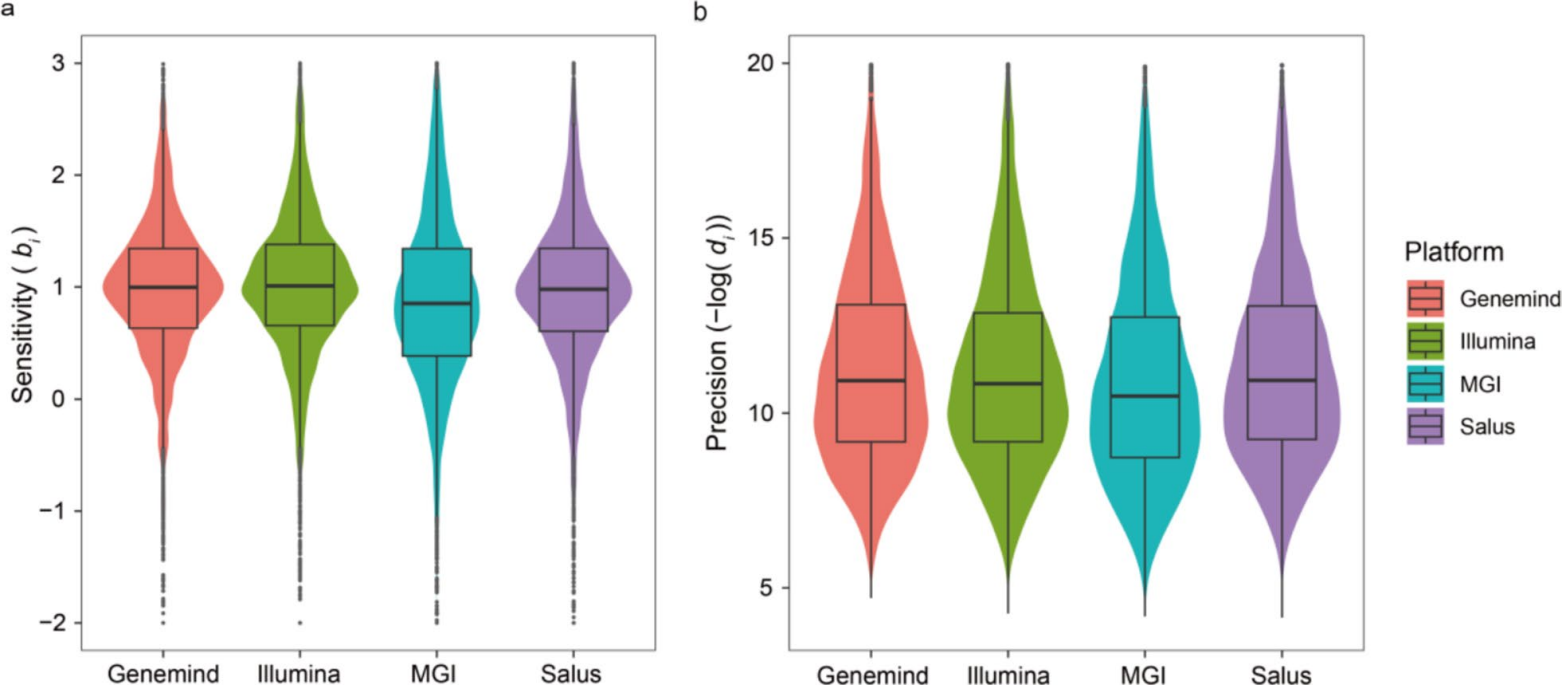
Measurement of sensitivities and precision across all four platforms: (a) sensitivity and (b) precision, the vertical axis represents negative log-transformed di

To evaluate variation in methylation signals across the four platforms, we first extracted platform-specific DMCs by performing one-to-many comparisons between platforms. For Genemind, Illumina, MGI, and Salus, there were 829, 1389, 109859, and 895 DMCs, respectively. Given that MGI had exhibited hypermethylation compared to the other platforms, it is unsurprising that MGI showed more DMCs than the others. Using Peters’ row-linear model [13], we evaluated the sensitivity and precision of these platforms. Larger *b*_*i*_ values correspond to higher sensitivity, while smaller *d*_*i*_ values correspond to higher precision. The median of sensitivities (*b*_*i*_) for Genemind, Illumina, MGI, and Salus were 1.00, 1.02, 0.87, and 0.99, respectively, and the median of precision (-log*d*_*i*_) were 11.03, 10.96, 10.57, and 11.06, respectively. The results indicated that cross-platform sensitivity and precision showed similar marginal medians for Genemind, Illumina, and Salus, while being lower for MGI (Fig. 6). Notably, the differences in both sensitivity and precision among the four platforms are minimal.

**Fig. 7:**
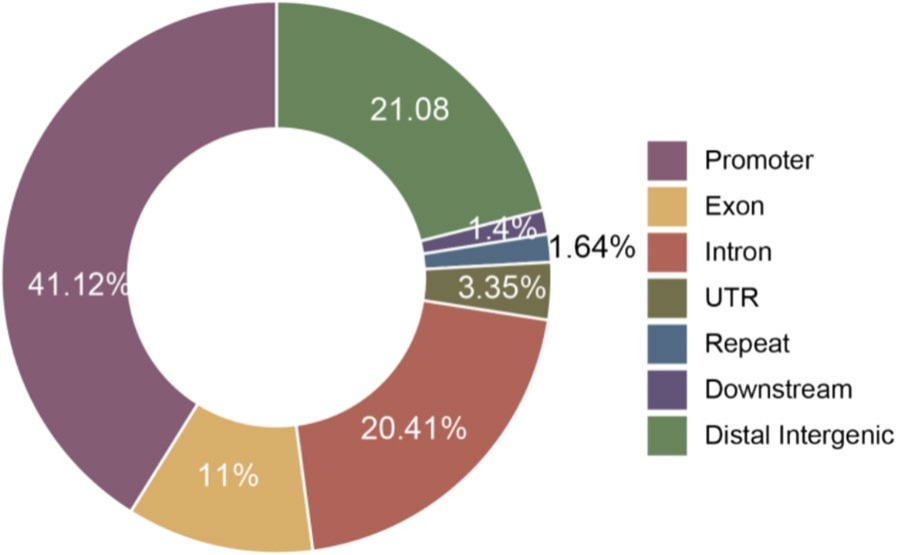
Genomic distribution of inter-platform discordant CpGs

To investigate the characteristics of these discordant CpGs, we annotated them to genomic elements. Annotation results revealed that the distribution of these DMCs was consistent with that of the overall CpG set, with the vast majority localized to gene regulatory regions (e.g., promoters) and gene bodies (Fig. 7). Notably, no significant enrichment of these discordant CpGs was observed in low-complexity regions.

## Discussion

The results of this study clearly show the differences among the four sequencing platforms. The Salus platform stands out in terms of sequencing quality, alignment rate, and data efficiency. The similar CpG detection rates and methylation level consistency among the platforms Genemind, Illumina, and Salus suggest that datasets generated by these three platforms can be used for integrated analysis in applications where these metrics are crucial, provided that the methylation level differences can be tolerated. However, the lower CpG detection rate and higher methylation level deviation of the MGI platform may limit its application in some scenarios. The deviation of the MGI platform may be attributed to its different sequencing principle. When integrating data from multiple platforms, it is essential to consider these platforms specific differences to ensure the accuracy and reliability of the final data analysis. One limitation of this study is the relatively small sample size, which may affect the generalizability of the results. Future research with a larger number of samples and different sample types is needed to further validate these findings.

## Conclusion

This comparative analysis of four sequencing platforms provides a comprehensive understanding of their performance characteristics. Each platform has its own advantages and disadvantages, and the choice of platform should be based on the specific requirements of the research or application. When considering multi-platform data integration, the differences in methylation levels and CpG detection rates should be carefully addressed.

Sequencing quality and data efficiency: The Salus platform distinguishes itself with the highest mapping rate (83.0%–83.4%) and effective depth (335–343X), coupled with a low duplication rate (14.3%–15.2%)—outperforming Genemind, Illumina, and MGI in these critical metrics. This makes Salus particularly suitable for studies requiring high data efficiency and minimal PCR bias, such as low-input cfDNA methylation analysis. Illumina and Genemind show comparable Q30 scores (91.7%–92.0% and 88.6%–89.1%, respectively) and BSRate (0.994), confirming their reliability for standard methylation studies. MGI exhibits the highest Q30 (95.2%–95.5%) among all platforms, reflecting exceptional base-calling accuracy, but lags in mapping rate (77.2%–78.5%) and effective depth (257–274X).

CpG detection capability: Genemind, Illumina, and Salus achieve consistent and high CpG detection rates (96.3%–96.9% at 1X coverage, > 90% at 5X coverage), enabling comprehensive capture of targeted CpG sites. In contrast, MGI shows a significantly lower CpG detection rate (91.2%–91.9% at 1X coverage, < 80% at 5X coverage)—a 17.3% reduction compared to the other three platforms at 5X coverage. This limitation may restrict MGI’s utility for studies requiring deep coverage of rare or low-abundance CpG loci, such as tumor-specific methylation biomarkers in liquid biopsies.

Methylation level consistency: A critical finding is the substantial discrepancy in methylation levels between MGI and the other three platforms. Genemind, Illumina, and Salus exhibit highly consistent mean methylation levels (44.6–46.6%) with only 15.9% of DMCs (|delta|> 0.05) among them, supporting their integration analysis in multi-platform studies. In contrast, MGI shows a markedly higher mean methylation level (66.8%) and 29.8% DMCs compared to the other platforms—with 26.6% of these DMCs being hypermethylated. This deviation, likely attributed to MGI’s unique cPAS sequencing principle and DNB library preparation, indicates that MGI data should not be directly integrated with data from bridge PCR-based platforms (Genemind, Illumina, Salus) without prior normalization.

This study addresses a critical gap in methylation research by offering objective, data-driven guidance for platform selection. While the small sample size (three cfDNA samples) is a limitation, the standardized library preparation (single library split into four sub-libraries) and unified analytical pipeline minimize experimental variability, ensuring the reliability of our comparative findings. Our findings offer valuable guidance for researchers and laboratories in making informed decisions regarding sequencing platform selection.

## Data Availability

All raw sequencing data generated in this study have been deposited in the Genome Sequence Archive database under the accession number PRJCA054612 (https://ngdc.cncb.ac.cn/bioproject/browse/PRJCA054612).
